# Is Mallein a Specific as a Diagnostic Agent?

**Published:** 1896-08

**Authors:** G. Leo HagenBurger


### ABSTRACTS FROM FOREIGN JOURNALS.

UNDER THE DIRECTION OF

J. PRESTON HOSKINS, PRINCETON, N. J.

IS MALLEIN A SPECIFIC AS A DIAGNOSTIC AGENT? By G. Leo HagenBurger, M.S., D.V.S. All of you are familiar with the use of the above in diagnosticating farcy or glanders, especially in its latency, so I will refrain from going into any details relating to its virtue in diagnosticating and treating the disease.

Having had an opportunity (some six years ago in New York City with a large railway company) to see quite a number of horses so affected, I recalled the theories of Senner on immunity of contagious diseases, and experimented with the serum of blood taken from cattle and dogs.

The injections at that time gave me little or no satisfaction; want of time and proper facilities compelled me to abandon it. Some sixteen months ago I had occasion to see a few cases in Brooklyn with some other practitioners, which were typical cases of glanders. Others I had seen in Maspeth (Queens County); another near Freeport, L. I. The following results show that mallein is *not the only specific in producing* a rise of temperature after injections:

Extract was of blood taken from a steer, ʒj diluted in ℥iv of water, and injected under the skin, the usual time allowed, and temperature then taken, showed a rise from 2° to 3° and 4° in all cases so injected. The three animals on post-mortem showed extensive lesions of the disease in the various organs. One horse injected with mallein four days after the first injection (extract of blood), and the same febrile symptoms appeared.

I have injected about ten horses in good health with the above, and no symptoms of any kind was noticed.

Then pursuing a different way, namely, using a more homogeneous product of the horse, an extract of bacillus coli communis, which was prepared by a friend of mine in a chemical laboratory.

One cubic centimetre being injected in a suspicious case, which was not typical, an examination showed a marked rise of temperature. The same animal two weeks later (after having had a purgative and starvation diet), being injected with mallein, reacted in the same way, and was consequently destroyed, and proved to be glandered. This leads me to assume that mallein is not a specific, and that we can get reactions from different heterogeneous bacteria in the form of an extract to diagnosticate the latency of the disease. If some of my brethren have an occasion to try it, I would like to hear the result, as the number of my cases has been limited, and I would therefore invite the aid of some gentlemen interested in this particular field.

